# No Evidence for Trade-Offs Between Lifespan, Fecundity, and Basal Metabolic Rate Mediated by Liver Fatty Acid Composition in Birds

**DOI:** 10.3389/fcell.2021.638501

**Published:** 2021-03-29

**Authors:** Sampath A. Kumar, Tomáš Albrecht, Ondřej Kauzál, Oldřich Tomášek

**Affiliations:** ^1^Institute of Vertebrate Biology, Czech Academy of Sciences, Brno, Czechia; ^2^Department of Botany and Zoology, Faculty of Science, Masaryk University, Brno, Czechia; ^3^Department of Zoology, Faculty of Science, Charles University, Prague, Czechia; ^4^Department of Ecology, Faculty of Science, Charles University, Prague, Czechia

**Keywords:** membrane pacemaker hypothesis, life-history trade-offs, pace-of-life syndromes, membrane unsaturation, evolution of longevity, ageing, aging, senescence

## Abstract

The fatty acid composition of biological membranes has been hypothesised to be a key molecular adaptation associated with the evolution of metabolic rates, ageing, and life span – the basis of the membrane pacemaker hypothesis (MPH). MPH proposes that highly unsaturated membranes enhance cellular metabolic processes while being more prone to oxidative damage, thereby increasing the rates of metabolism and ageing. MPH could, therefore, provide a mechanistic explanation for trade-offs between longevity, fecundity, and metabolic rates, predicting that short-lived species with fast metabolic rates and higher fecundity would have greater levels of membrane unsaturation. However, previous comparative studies testing MPH provide mixed evidence regarding the direction of covariation between fatty acid unsaturation and life span or metabolic rate. Moreover, some empirical studies suggest that an n-3/n-6 PUFA ratio or the fatty acid chain length, rather than the overall unsaturation, could be the key traits coevolving with life span. In this study, we tested the coevolution of liver fatty acid composition with maximum life span, annual fecundity, and basal metabolic rate (BMR), using a recently published data set comprising liver fatty acid composition of 106 avian species. While statistically controlling for the confounding effects of body mass and phylogeny, we found no support for long life span evolving with low fatty acid unsaturation and only very weak support for fatty acid unsaturation acting as a pacemaker of BMR. Moreover, our analysis provided no evidence for the previously reported links between life span and n-3 PUFA/total PUFA or MUFA proportion. Our results rather suggest that long life span evolves with long-chain fatty acids irrespective of their degree of unsaturation as life span was positively associated with at least one long-chain fatty acid of each type (i.e., SFA, MUFA, n-6 PUFA, and n-3 PUFA). Importantly, maximum life span, annual fecundity, and BMR were associated with different fatty acids or fatty acid indices, indicating that longevity, fecundity, and BMR coevolve with different aspects of fatty acid composition. Therefore, in addition to posing significant challenges to MPH, our results imply that fatty acid composition does not pose an evolutionary constraint underpinning life-history trade-offs at the molecular level.

## Introduction

The fatty acid (FA) composition of biological membranes has a strong influence on many cellular processes (Grecco et al., 2011). This fact has sparked the development of hypotheses that propose that the membrane FA composition is a key molecular adaptation underpinning the evolution of metabolic rates, ageing, and life span (Hulbert and Else, 1999; Pamplona et al., 2002; Hulbert, 2010). The most prominent of these hypotheses is the membrane pacemaker hypothesis (MPH), which proposes that the level of membrane FA unsaturation could act as a pacemaker of metabolism and ageing, thereby determining species-specific life span (Hulbert and Else, 1999; Hulbert, 2008). More specifically, MPH proposes two interacting molecular mechanisms linking membrane FA composition to the rates of metabolism and ageing.

First, the incorporation of unsaturated FA (i.e., those containing at least one double bond) into biological membranes enhances membrane fluidity (Jaureguiberry et al., 2014) and the activity of membrane proteins, which may enable higher cellular and organismal metabolic rates (Hulbert, 2008). Mechanistically, the increase in fluidity with unsaturation is attributed to the presence of *cis* double bonds that bend the acyl chains of unsaturated FA, resulting in greater interchain distances and, consequently, in the decrease in van der Waals force of intermolecular attraction (Hulbert and Else, 1999). The addition of polyunsaturated FA (PUFA) also modifies the spatial distribution of lipid rafts (i.e., the relatively ordered and rigid membrane lipid regions), pushing them closer to each other and stabilising raft-associated proteins (Shaikh et al., 2015). Both membrane fluidity and the effect of PUFA on the spatial distribution of lipid rafts may enhance the activity of membrane proteins important for energetic metabolism, such as glucose transporter proteins (Weijers, 2016) and Na^+^/K^+^ ATPases (Lingwood et al., 2005; Welker et al., 2007), respectively. These effects at the molecular level may eventually lead to higher metabolic rates at the cellular and organismal level (Hulbert, 2008). Higher metabolic rates are often associated with increased generation of free radicals, which induce oxidative damage to vital cellular components (Perez-Campo et al., 1998; da Costa et al., 2016). Hence, in addition to enhanced metabolic rates, higher membrane unsaturation can increase the rate of senescence according to MPH because the accumulation of oxidative damage has been proposed as a major cause of senescence by the free-radical theory of ageing (Barja, 2013).

Second, owing to the presence of highly reactive hydrogen atoms in their double bonds, unsaturated FA are known to be more prone to oxidative damage by free radicals and other reactive oxygen species (ROS; Holman, 1954; Hulbert, 2005). Based on this knowledge, MPH suggests that the level of membrane unsaturation influences senescence rate not only through increased ROS generation accompanying fast metabolic rates but also through susceptibility of unsaturated membranes to oxidative damage (Pamplona et al., 2002; Pamplona and Barja, 2011).

The appeal of MPH lies in its potential to provide a simple mechanistic explanation of the central paradigm of the life-history theory, namely the physiological trade-off between metabolic rate and fecundity on one side and the rate of senescence and longevity on the other (Stearns, 1989, 1992; Ricklefs and Wikelski, 2002). In other words, MPH implies that membrane unsaturation may be a major molecular mechanism constraining the evolution of life histories (or pace of life) along the fast–slow continuum with highly fecund, short-lived species on one end and long-lived species with low fecundity on the opposite end (Williams et al., 2010; Jimenez et al., 2014; Gaillard et al., 2016).

However, the causal role of ROS in senescence has been questioned (Lapointe and Hekimi, 2010; Speakman and Selman, 2011), challenging both the free-radical theory of ageing and MPH although such challenges have been dismissed as misconceptions by defenders of those two hypotheses (Barja, 2013) or may have resulted from methodological pitfalls (Munro and Pamenter, 2019). Moreover, the empirical support for MPH has been mixed so far, showing negative, no, or positive correlation between membrane unsaturation and life span or metabolic rate although studies focused on life span provide relatively more support for MPH (reviewed in Calhoon et al., 2015; Jové et al., 2020). It is also worth noting that many early studies supporting MPH are based on limited sample sizes (often comparing one short-lived mammal with one longer-lived avian species) or do not control for the confounding effects of body mass and phylogenetic non-independence of different species (Naudí et al., 2013; Valencak and Azzu, 2014; Calhoon et al., 2015; Bozek et al., 2017; Jové et al., 2020). Statistical control for the confounding effect of body mass is particularly important as the observed relationships could potentially arise due to the correlation of membrane unsaturation with body mass while not being associated with longevity directly (Speakman, 2005b; Valencak and Azzu, 2014). When controlling for body mass and phylogenetic autocorrelation, comparative studies using sufficient numbers of mammalian or avian species found no (Valencak and Ruf, 2007) or even a positive (Galván et al., 2015) relationship between FA unsaturation and maximum life span.

Several lines of experimental evidence further suggest that the association between FA unsaturation and susceptibility to oxidative stress may not be as straightforward as commonly assumed. For example, the peroxidation rate of FA in an aqueous environment may not reflect their degree of unsaturation (Visioli et al., 1998). In cell cultures and plants, PUFA have even been reported to efficiently scavenge ROS and protect other biomolecules from oxidative damage (Richard et al., 2008; Mène-Saffrané et al., 2009). The ROS-scavenging effect was positively related to the degree of PUFA unsaturation with n-3 PUFA being the most effective ones. Such observations led to the hypothesis that membranes rich in PUFA may act as structural antioxidants (Richard et al., 2008; Mène-Saffrané et al., 2009; Farmer and Mueller, 2013; Schmid-Siegert et al., 2016). Such an antioxidant function of PUFA could provide a possible explanation for the positive relationship between maximum life span and the degree of liver FA unsaturation reported by a recent phylogenetic comparative study on 107 bird species (Galván et al., 2015). However, despite the mixed support for MPH and the emergence of alternative hypotheses about PUFA properties (Richard et al., 2008; Mène-Saffrané et al., 2009; Farmer and Mueller, 2013; Schmid-Siegert et al., 2016), it is still widely assumed that long-lived species evolve membranes with low FA unsaturation (Schroeder and Brunet, 2015; Johnson and Stolzing, 2019; Papsdorf and Brunet, 2019; Jové et al., 2020).

To further complicate matters, a phylogenetic comparative study on 42 mammalian species found a negative relationship between maximum life span and n-3/n-6 PUFA ratio (Valencak and Ruf, 2007), which contrasts with the experimental evidence purporting antioxidant properties to n-3 PUFA. In addition, the length of the FA carbon chain could be a key trait coevolving with longevity as suggested by a recent comparative study on birds, which reported a positive relationship between life span and length of liver FA (Galván et al., 2015). However, such a positive relationship is in contrast with a mammalian comparative study, showing lower concentrations of long-chained FA in the blood plasma of long-lived species (Jové et al., 2013). It is unclear whether such contrasting results arise from physiological differences between taxa or because of different choices of tissues or statistical approaches among the studies. Consequently, the importance of membrane FA composition for life span or metabolic rate evolution remains an unresolved question (Calhoon et al., 2015).

Comparative studies may provide important insights into physiological and biochemical mechanisms modulating longevity and metabolic rates, considering the great variation in life-histories among various species even when body mass is accounted for Munro and Pamenter (2019). Here, we combine recently published data on liver FA of 106 European bird species (Galván et al., 2015) with published data on life-history traits (Fransson et al., 2017; Storchová and Hořák, 2018) and basal metabolic rate (BMR; McNab, 2009) to examine the links between FA composition and maximum life span, annual fecundity, or BMR. We include annual fecundity because reproduction is a highly energy-demanding process associated with elevated metabolic rates (Drent and Daan, 1980). To our knowledge, the coevolution of fecundity with FA composition has never been tested before even though it logically follows from the hypothesis that FA unsaturation should act as a pacemaker for metabolism and, hence, pace of life. The relationship between FA composition and maximum life span was analysed in the original study (Galván et al., 2015). The authors suggest that long life span evolves with higher proportions of long-chain and monounsaturated FA (MUFA), lower proportion of PUFA, and, surprisingly, higher overall level of unsaturation. They further observed that long life span was related to lower anti-inflammatory index, which is a ratio of anti-inflammatory FA to pre-inflammatory arachidonic acid. Nevertheless, Galván et al. (2015) relate life span to the first component of partial least squares regression, which, in addition to FA indexes, also include the effect of body mass and phylogeny. Hence, it is unclear to what extent the results of the original study may be affected by those confounding effects.

We employ Bayesian hierarchical (mixed effect) models, which allow us to perform beta regression within a phylogenetic context (Douma and Weedon, 2019) while controlling for the confounding effects of body mass and phylogeny. Beta regression is the preferred method to analyse proportional data, such as the proportions of FA because the commonly applied Gaussian regression of logit or square root–transformed data may yield biased estimates. The potential for such a bias is particularly high when the proportions are close to zero or one (Douma and Weedon, 2019) as is the case with many FA (Galván et al., 2015). Because differences in FA metabolism may evolve between sedentary and migratory species – either resulting from metabolic adaptations to long-distance flight (Jenni and Jenni-Eiermann, 1998; Guglielmo, 2010) or due to wintering in different environments (Calhoon et al., 2014; Jimenez et al., 2014) – we further include migratory behaviour as a covariate in the models. Additionally, sedentary and migratory species breeding in the temperate zone are also known to differ in their life-histories (Mönkkönen, 1992; Soriano-Redondo et al., 2020) and physiological components of pace of life (McNab, 2009; Gavrilov, 2014; Tomasek et al., 2019).

While statistically controlling for interspecific variation in body mass, migratory behaviour, and phylogeny, we aimed (i) to test whether maximum life span, annual fecundity, and BMR are associated with the aspects of FA composition proposed previously (i.e., FA unsaturation, n-3 PUFA/total PUFA proportion, average chain length, and anti-inflammatory index) and (ii) to carry out a detailed analysis exploring the associations between life-history traits and individual fatty acids.

## Materials and Methods

### FA Data

We obtained data on the total liver FA composition of 106 European bird species encompassing 15 taxonomic orders from Galván et al. (2015). The original data set consisted of 107 species, but we excluded common cuckoo (*Cuculus canorus*), which is an obligate brood parasite with indefinable clutch size and complete lack of parental care. The authors utilised liver tissues of free-living birds collected in Denmark to extract total lipids, followed by conversion of FA to FA methyl esters and quantification by gas chromatography. The molar proportions of individual FA were given as the mean values per species.

In addition to the proportions of individual FA, we further used the following set of FA indices available in the original data set: the proportions of saturated FA (SFA), MUFA, and PUFA; the proportion of n-3 PUFA to total PUFA (hereafter, n3 PUFA/total PUFA); average chain length (ACL); double bond index (DBI); peroxidizability index (PI); and anti-inflammatory index (AI). We calculated n-3 PUFA/total PUFA as an alternative to n-3/n6 PUFA ratio as analysing ratios in regression models is challenging (Sollberger and Ehlert, 2016). ACL indicates the average length of the FA carbon chain based on the proportional representation of individual FA. DBI gives the mean number of double bonds per 100 fatty acids. PI reflects the susceptibility of fatty acids to oxidative damage and is calculated as follows: PI = [(0.025 × Σmol% monoenoic) + (1 × Σmol% dienoic) + (2 × Σmol% trienoic) + (4 × Σmol% tetraenoic) + (6 × Σmol% pentaenoic) + (8 × Σmol% hexaenoic)] (Holman, 1954; Galván et al., 2015). AI is the percentage ratio of FA with anti-inflammatory properties (20:3n-6, 20:5n-3, 22:6n-3) to pre-inflammatory arachidonic acid (20:4n-6) (Galván et al., 2015).

### Life-History and BMR Data

To control for the confounding effect of body mass (Speakman, 2005b), we used data on body mass from the original FA data set (Galván et al., 2015). We obtained maximum life span estimates predominantly from EURING (Fransson et al., 2017). Only for the water pipit (*Anthus spinoletta*), which had no maximum life span estimate in EURING, we used the corresponding record from AnAge longevity database (de Magalhães and Costa, 2009). We also extracted the number of recoveries of each species from EURING as capture effort is positively correlated with estimates of species maximum life span (Møller, 2008; Tomasek et al., 2019). To calculate annual fecundity (number of eggs laid per year), we utilised data on clutch size and number of clutches per year from Storchová and Hořák (2018).

Data on BMR came from the data set compiled by McNab (2009) except for the white-throated dipper (*Cinclus cinclus*) for which the value was obtained from a separate study (Bryant and Newton, 1994). BMR values were reported in kJ/h and adhered to the standard set of conditions necessary for metabolic rate measurements to be regarded as BMR (McKechnie and Wolf, 2004; McNab, 2009).

### Migration Distance

Migration distance was calculated as a distance in thousands of kilometres between the centroid of the Jutland part of Denmark (original sampling location of data used in Galván et al., 2015) and the centroid of non-breeding distribution of each species using the haversine formula. Map data were obtained from BirdLife International and Handbook of the Birds of the World (2018). Centroids were calculated based on species distributions limited to Europe and Africa, using the ArcGIS 10.3 software (Esri, Redlands, CA, United States). The migration distance was set to zero in sedentary species.

### Phylogenetic Data

Species phylogenetic non-independence is a form of autocorrelation due to shared ancestry (some species are more closely related to each other than to other species) and needs to be controlled for statistically to avoid biased parameter estimates (Speakman, 2005b). To control for the effect of phylogenetic non-independence, we used the most complete molecular phylogeny of extant bird species to date^1^ based on the phylogenetic hypotheses of Hackett et al. (2008) and Jetz et al. (2012). For the 106 species included in our study, we downloaded 10,000 phylogenetic trees and generated a consensus tree using the TreeAnnotator tool implemented in the BEAST 2.6.3 software (Bouckaert et al., 2019).

### Statistical Analysis

We analysed the data using Bayesian hierarchical models implemented in the brms package (Bürkner, 2018) in R 4.0.3 software (R Core Team, 2020). We used this approach rather than the phylogenetic least squares because it allowed us to control for species-level phylogenetic non-independence while applying beta regression, which is the preferred method for continuous proportional data (Douma and Weedon, 2019). In addition, Bayesian inference does not need corrections for multiple testing because it estimates the probability of an effect of interest given the prior belief and the observed data and, unlike the frequentist framework, does not make assumptions about the distribution of *p* values in theoretical repetitions of an experiment (Berry and Hochberg, 1999; Gelman et al., 2012; Sjölander and Vansteelandt, 2019). Nevertheless, we follow the suggestion of Sjölander and Vansteelandt (2019) and adjust for multiple testing informally by discussing the effects while considering their dependence (Figure 2) and relative support (Figure 3).

In the beta regression models, we fitted proportions of the individual FA, SFA, MUFA, PUFA, or n-3 PUFA/total PUFA as response variables. In addition, we used Gaussian regression to model non-proportional response variables, namely ACL, DBI, PI, and log-transformed AI.

We fitted four types of regression models differing in the fixed-effect structure. First, to estimate the effect of body mass, we fitted simple phylogenetic regression models with body mass as the sole predictor (Supplementary Table 1). Second, to analyse the association of FA composition with life span, the models contained maximum life span as the focal predictor (Supplementary Table 2). These models further included body mass, number of recoveries, and migration distance among the predictors to control for their effects. Third, to analyse the association of FA composition with fecundity, we fitted models with annual fecundity, body mass, and migration distance as predictors (Supplementary Table 3). The fourth set of models included total BMR along with body mass as the predictors (Supplementary Table 4). We log-transformed body mass, annual fecundity, BMR, and the number of recoveries before fitting them in the models. We also standardised (*z*-transformed) each predictor and non-proportional response variable (ACL, DBI, PI, log-transformed AI) by subtracting its mean from the raw values and dividing them by standard deviation of the variable. Phylogeny was included in all the models as a random effect (Garamszegi, 2014). We used default priors calculated by the brms R package and ran the models in 25 chains, each with 6,000 iterations, 2,000 burn-in, and thinning set to 10. Potential scale reduction factor was <1.01 in all cases, indicating good model convergence (Gelman and Rubin, 1992).

We present resulting estimates as posterior means together with their equal-tailed 95% credible intervals based on quantiles (Bürkner, 2017). In the Bayesian framework, the 95% credible interval represents a range of values that, given the observed data, contain the true effect value with 95% probability. We consider the support for an effect to be significant if 95% credible intervals do not contain zero (Hespanhol et al., 2019).

## Results

### Interspecific Variation in Liver FA Composition

**FIGURE 1:**
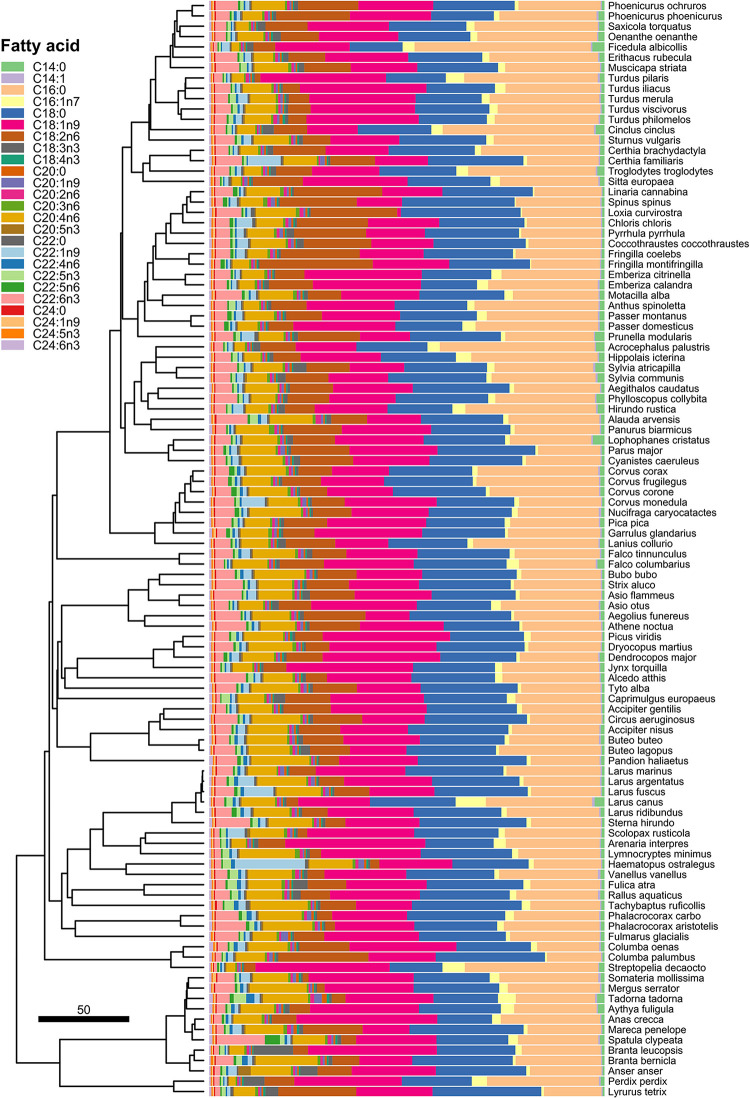
Interspecific variation in relative proportions of the individual liver FA.

The abundance and interspecific variation in liver FA is visualised in Figure 1. The most abundant liver FA according to their median values were palmitic acid (C16:0; 23.3%), stearic acid (C18:0; 22.2%), oleic acid (C18:1n9; 21.1%), linoleic acid (C18:2n6; 9.3%), arachidonic acid (C20:4n6; 8.5%), docosahexaenoic acid (C22:6n3; 3.5%), and palmitoleic acid (C16:1n7; 1.3%). Medians of all the other FA were lower than 1%.

**FIGURE 2:**
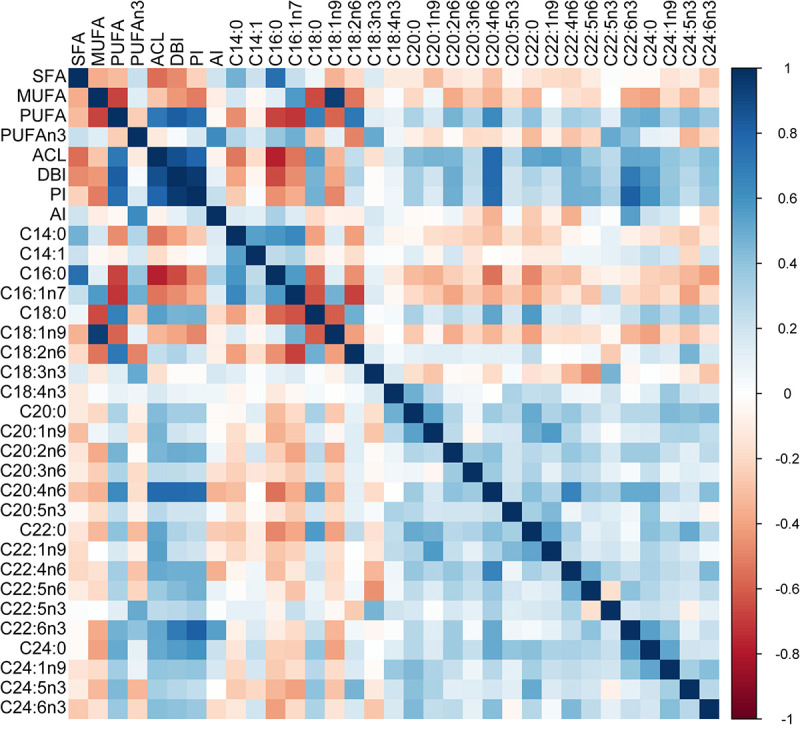
Pairwise correlations between residuals of FA indices and individual FA. Correlations were calculated using residuals from phylogenetic generalised least squares regression on body mass. The proportional variables were logit-transformed, and AI was log-transformed before the analysis. SFA – saturated fatty acids, MUFA – monounsaturated fatty acids, PUFA – polyunsaturated fatty acids, DBI – double bond index, PI – peroxidizability index, ACL – average chain length, AI – anti-inflammatory index.

**FIGURE 3:**
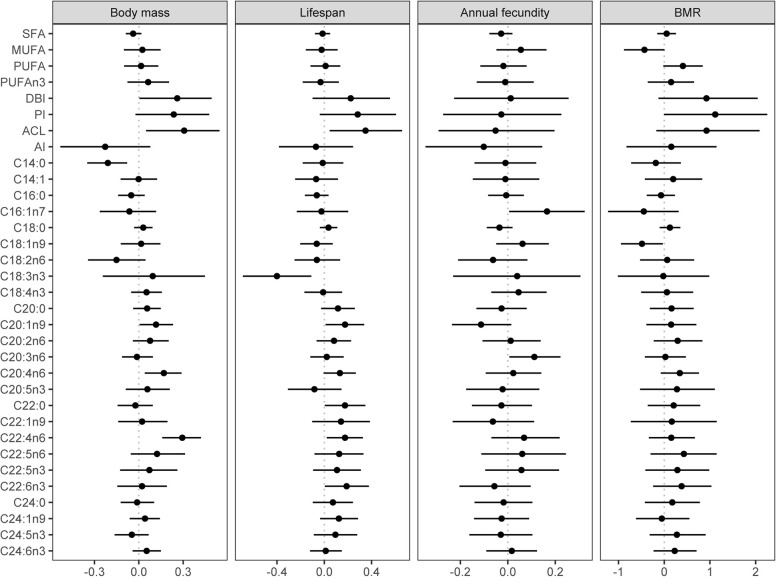
Effects of body mass, life span, annual fecundity, and BMR on liver FA composition. Shown are average marginal effects from Bayesian phylogenetic models and their 95% credible intervals. The 95% credible interval represents a range of values that, given the observed data, contain the true effect value with 95% probability. We consider the effect to be significantly supported if the 95% credible interval do not contain zero. Effect of body mass is from a model with body mass as a sole fixed-effect predictor. The effect of life span was controlled for body mass, number of recoveries, and migration distance. The effect of annual fecundity was controlled for body mass and migration distance. The effect of BMR was controlled for body mass. Body mass, life span, number of recoveries, annual fecundity, BMR, and anti-inflammatory index were log-transformed. All the non-proportional response and predictor variables were standardised by *z*-transformation. Beta regression and Gaussian regression were used for proportional and non-proportional data, respectively. SFA – saturated fatty acids, MUFA – monounsaturated fatty acids, PUFA – polyunsaturated fatty acids, DBI – double bond index, PI – peroxidizability index, ACL – average chain length, AI – anti-inflammatory index.

Index of overall FA unsaturation (DBI) and PI were strongly positively correlated to ACL and PUFA (Figure 2). At the level of individual FA, DBI, and PI showed the strongest positive correlations with C20:4n6 and C22:6n3 and the strongest negative correlation with C16:0. ACL was most strongly associated with C20:4n6. Besides this, C18:0 and all the FA with 20 or more carbons in their chains contributed to ACL as all of them showed positive correlations with ACL. In contrast, ACL was negatively correlated with C14:0, C16:0, C16:1n7, and C18:1n9. Moreover, the pattern of pairwise correlations between individual FA suggests that ACL is the primary axis of variation. We only show correlations of residuals from a phylogenetic generalised least squares regression on body mass in Figure 2 because there was no difference between correlations based on raw values. This indicates that the correlation structure of liver FA is independent of body mass and phylogeny.

### Scaling of Liver FA Composition With Body Mass

With regards to the FA indices, only DBI and ACL exhibited a positive covariation with body mass (Figure 3; Supplementary Table 1). Body mass was not significantly associated with most individual FA. Only C14:0 showed a negative association with body mass, whereas C20:1n9, C20:4n6, and C22:4n6 showed a negative association.

### Liver FA Composition and Life Span

In contradiction to MPH, our analysis did not reveal a significant relationship between SFA, MUFA, PUFA, DBI, or PI and maximum life span while controlling for confounding effects of body mass, migratory behaviour, and phylogeny (Supplementary Table 2; Figure 3). Such a result indicates that species life span is not determined by FA unsaturation. There was also no association of life span with n-3 PUFA/total PUFA or anti-inflammatory index. The only integrative component of FA composition that did correlate in a significant manner with maximum life span was ACL (Figure 3).

At the level of individual FA, long life span was associated with low levels of C18:3n3 and high levels of C20:1n9, C22:0, C22:4n6, and C22:6n3. There was also weak support for a positive covariation of maximum life span with 20:4n6, but the 95% credible intervals of the estimate marginally contained zero (Figure 3).

### Liver FA Composition and Annual Fecundity

Our analysis did not reveal a significant correlation between any of the FA composition indices and annual fecundity. The only individual FA that were associated with annual fecundity were C16:1n7 and C20:3n6, both exhibiting positive association (Figure 3; Supplementary Table 3).

### Liver FA Composition and BMR

Basal metabolic rate was not significantly associated with any of the indices of the liver FA composition although there were marginally non-significant trends toward a positive covariation with PUFA, DBI, and PI and a negative covariation with MUFA. Among the individual FA, only C18:1n9 showed a significant association in the negative direction (Figure 3; Supplementary Table 4).

## Discussion

Our comparative analysis does not support the MPH as a whole because none of the key indices of FA unsaturation (DBI, PI, SFA, MUFA, and PUFA) covaried with maximum life span, BMR, or annual fecundity when controlled for the confounding effects of body mass, migration behaviour and phylogeny. Contrary to the previous analysis of the same data set (Galván et al., 2015), our results do not support the conclusion that long life span evolves with high MUFA and low PUFA content, which is commonly considered to be one of the mechanisms underpinning negative covariation between unsaturation and longevity (Schroeder and Brunet, 2015; Johnson and Stolzing, 2019; Papsdorf and Brunet, 2019; Jové et al., 2020). Moreover, maximum life span tended to positively covary, although non-significantly, with DBI and PI in our study, providing strong evidence against the prediction that long-lived species evolve membranes with low FA unsaturation.

Although the early comparative studies support a negative relationship between FA unsaturation and longevity, those studies are mostly based on only a low number of species, sometimes comparing one short-lived mammal species with one longer-lived bird species, and do not control for the confounding effects of body mass and phylogenetic non-independence (Naudí et al., 2013; Valencak and Azzu, 2014; Calhoon et al., 2015; Bozek et al., 2017; Jové et al., 2020). Modern phylogenetic comparative studies utilising larger sample sizes and controlling for body mass and phylogeny found no (Valencak and Ruf, 2007) or even positive (Galván et al., 2015) association between FA unsaturation and maximum life span. Similarly, the positive associations between FA unsaturation and BMR or calcium ATPase in muscles have not been supported in phylogenetic comparative studies on mammals and fish, respectively (Valencak and Ruf, 2007; Gonzalez et al., 2015). Moreover, one of the fundamental assumptions of MPH coming from the original rate-of-living theory (Pearl, 1928) – i.e., the negative correlation between metabolic rates and life span – has been refuted, at least at the interspecific level, by large comparative studies controlling for body mass and phylogeny (Speakman, 2005a; de Magalhães et al., 2007; Furness and Speakman, 2008; Møller, 2008). Our results fit into such emerging comparative evidence against MPH, specifically against the prediction that evolution of long life span is underpinned by low FA unsaturation. Nevertheless, marginally non-significant positive association between PI and BMR may provide some, although very weak, support for the second prediction of MPH, namely that higher FA unsaturation enhances metabolic rates (Hulbert and Else, 1999).

It is important to note, however, that the total liver lipids analysed in our study and that by Galván et al. (2015) also comprise lipids other than membrane phospholipids, such as triglycerides derived from the diet. Hence, caution should be exercised when interpreting the observed patterns as reflecting variation in membrane FA composition. However, it seems reasonable to assume that phospholipid FA contribute considerably to the patterns because phospholipids represent the majority of lipids in the avian liver (Grande and Prigge, 1970; Galván et al., 2015). Moreover, a phylogenetic analysis comparing the composition of muscle phospholipids across mammals similarly reported a lack of an association between life span and FA unsaturation or the contents of SFA, MUFA, and PUFA (Valencak and Ruf, 2007), suggesting that the absence of such relationships in our study is probably not due to a confounding effect of dietary triglycerides.

Our analysis does not support several conclusions of the original study analysing the same data set (Galván et al., 2015), namely associations of maximum life span with proportions of MUFA and PUFA, anti-inflammatory index, or several individual FA, including C18:1n9, C18:2n6, C20:3n6, C22:1n9, and C22:5n6. Instead, we identified some other FA to be associated with maximum life span (C18:3n3, C20:4n6, C22:0, and C22:6n3). Such a discrepancy is most probably down to the different statistical approaches employed. The conclusion of the original study was based on the significant contributions of those indices and FA to the life span–predicting component of partial least square regression; however, that component integrated variation shared by all the FA indices, body mass, and phylogeny. It is, therefore, unclear as to what extent MUFA covaried with maximum life span directly. Because the conventional phylogenetic regression model used in our study is more straightforward to interpret, the discrepancies between the studies suggest potential pitfalls of the interpretation of individual predictors in partial least squares regression. Another factor potentially contributing to such discrepant conclusions could be that the original study used square-root transformation of proportional data, which may lead to biased estimates (Douma and Weedon, 2019). In contrast, we used beta regression in our study, which is the preferred statistical method for proportional data (Douma and Weedon, 2019).

In accordance with the conclusions of the original study (Galván et al., 2015), our results show a positive covariation between maximum life span and ACL, supporting the role of FA chain length in life span evolution. Considering the differences in statistical approaches explained above, our analysis provides an improved interpretation of the positive longevity–ACL relationship as being independent of the confounding effects of body mass and phylogeny. Our analysis of individual FA further indicates that this covariation was mainly driven by long life span being associated with low levels of α-linolenic acid (C18:3n3) and high levels of gondoic acid (C20:1n9), behenic acid (C22:0), adrenic acid (C22:4n6), and docosahexaenoic acid (DHA; C22:6n3). Nevertheless, except for the eicosapentaenoic acid (C20:5n3), all the other FA with 20 or more carbons in their chains probably contributed to the longevity–ACL relationship because their models show positive regression coefficients of life span although with 95% credible intervals containing zero. Taken together, these results suggest that longevity does not evolve with any particular type of FA as life span was positively associated with at least one long-chain FA of each type (i.e., SFA, MUFA, n-6 PUFA, and n-3 PUFA).

Fatty acid unsaturation was positively associated with maximum life span in the original study (Galván et al., 2015). Here, we observed a similar, although non-significant, trend. On one hand, such a positive covariation between life span and unsaturation is consistent with the hypothesis that membranes rich in long-chain PUFA function as structural antioxidants (Richard et al., 2008; Mène-Saffrané et al., 2009; Farmer and Mueller, 2013; Schmid-Siegert et al., 2016). On the other hand, the fact that all the types of long-chain FA, irrespective of their unsaturation, covaried positively with life span in our analysis indicates that unsaturation is not the key trait in life span evolution. Moreover, ACL showed a much stronger association with life span than DBI or PI in our study, suggesting that the positive covariation of unsaturation indices with life span more likely reflects a tight relationship between fatty acid unsaturation and chain length (Figure 2).

Another feature of FA composition proposed to be linked to life span evolution is the n-3/n-6 PUFA ratio (Valencak and Ruf, 2007). Specifically, long life span is reported to evolve with relatively smaller proportions of n-3 PUFA in muscle phospholipids across mammals (Valencak and Ruf, 2007). It is further suggested that a low level of DHA, a highly unsaturated n-3 PUFA, is particularly important for the evolution of long life span due to its high susceptibility to peroxidation (Pamplona et al., 1998; Hulbert, 2003). Our results do not support the importance of n-3/n-6 PUFA ratio, however, as the n-3 PUFA/total PUFA did not show any association with maximum life span. Moreover, DHA was significantly positively related to maximum life span in our analysis, which is contrary to the proposed negative correlation (Pamplona et al., 1998; Hulbert, 2003). The positive association between DHA and life span is intriguing and has not been observed in previous comparative studies, including the original study analysing the same data set using partial least squares analysis (Valencak and Ruf, 2007; Galván et al., 2015). Such a pattern provides other evidence against MPH. It is rather consistent with the hypothesis that membranes with long-chain PUFA act as structural antioxidants (Richard et al., 2008; Mène-Saffrané et al., 2009; Farmer and Mueller, 2013; Schmid-Siegert et al., 2016). As discussed, however, the positive association of DHA with life span may merely reflect the overall importance of long-chain FA irrespective of their unsaturation.

Docosahexaenoic acid was further proposed as an important pacemaker of metabolic rate (Hulbert and Else, 1999; Hulbert, 2003) based on its observed enhancing effect on the molecular activity of Na^+^/K^+^-ATPase (Turner et al., 2003). Our results do not support such a role of DHA at the interspecific level in birds as DHA was not significantly associated with BMR. This is in accordance with the findings of a previous comparative study in mammals, which also reported no relation between DHA and BMR (Valencak and Ruf, 2007). Hence, DHA is probably not an important molecular adaptation underpinning metabolic rate evolution.

Nevertheless, the main factor hypothesised to underpin a variation in metabolic rates is membrane unsaturation (Hulbert and Else, 1999). Our findings provide weak support for this hypothesis, revealing marginally non-significant positive covariation of BMR with DBI, PI, and PUFA and negative covariation with MUFA. These patterns contrast with the results reported by the Valencak and Ruf (2007) comparative study on mammals, which found no link between BMR and DBI, PUFA, or MUFA and even a negative association of BMR with PI. However, the high level of MUFA in species with low BMR observed in our analysis is in accordance with the recent study in dogs, demonstrating a decrease in cellular metabolic rate following an enrichment of fibroblasts with MUFA (Jimenez et al., 2020). It is important to note, however, that 95% credible intervals of our estimates marginally contained zero, and we performed a high number of comparisons in our analysis. Clearly, more phylogenetic comparative studies focused on FA composition of phospholipids are needed to validate the hypothesised macroevolutionary link between membrane unsaturation and metabolic rates.

In conclusion, our analysis presents significant challenges to the current hypotheses about the role of FA composition in life span and life-history evolution. We found no support for membrane unsaturation, MUFA/PUFA ratio, or n-3/n-6 PUFA ratio playing an important role in life span evolution as commonly assumed based on MPH (Hulbert, 2003; Schroeder and Brunet, 2015; Johnson and Stolzing, 2019; Papsdorf and Brunet, 2019; Jové et al., 2020). Our results rather support the importance of FA chain length for life span evolution as proposed by the previous study (Galván et al., 2015). Our contribution to such a conclusion lies in a clear demonstration that the positive longevity–ACL relationship is independent of body mass and phylogeny. We only found weak support for the second MPH prediction, namely that high membrane unsaturation promotes high metabolic rates, thereby functioning as a pacemaker of metabolic rates; however, the weakness of the effect prevents any firm conclusion in this regard. We also identified specific types of FA associated with maximum life span, annual fecundity, and BMR evolution that may represent promising avenues for future research. A striking finding of our study is that life span, fecundity, and BMR are each associated with different FA or FA indices. Not even a single FA showed significant association with more than one life-history trait or BMR. Such a pattern indicates that maximum life span, annual fecundity, and BMR coevolve with different aspects of liver FA composition. Such differences imply that liver FA composition does not pose molecular constraints underpinning life-history trade-offs.

## Data Availability Statement

The original contributions presented in the study are included in the article/Supplementary Material, further inquiries can be directed to the corresponding author.

## Ethics Statement

Ethical review and approval was not required for the animal study because publicly available data were only used in this study.

## Author Contributions

OT conceived and designed the study with input from TA. SK, OK, and OT collected and processed the data. SK and OT analysed data with input from TA. SK and OT wrote the manuscript with input from TA and OK. All authors approved the submitted version.

## Conflict of Interest

The authors declare that the research was conducted in the absence of any commercial or financial relationships that could be construed as a potential conflict of interest.

## Footnotes

1 BirdTree.org

## Abbreviations

ACL: average chain length
AI: anti-inflammatory index
DBI: double bond index
DHA: docosahexaenoic acid
MPH: membrane pacemaker hypothesis
PI: peroxidizability index.
