# Supplementary material for: No Evidence for Trade-Offs Between Lifespan, Fecundity, and Basal Metabolic Rate Mediated by Liver Fatty Acid Composition in Birds
Source: Front Cell Dev Biol. 2021 Mar 29;9:638501. doi: 10.3389/fcell.2021.638501 (PMC8045231; doi:10.3389/fcell.2021.638501)
Supplement: Supplementary file 1 [file Data_Sheet_1.docx]

Supporting Information for

**No evidence for trade-offs between lifespan, fecundity and basal metabolic rate mediated by liver fatty acid composition in birds fatty acids**

Sampath A. Kumar, Tomáš Albrecht, Ondřej Kauzál, Oldřich Tomášek

**Table S1**. Average marginal effects from body mass models. Values are effect estimates from Bayesian phylogenetic models with 95% credible intervals.

| **Response** | **Body mass** |
| --- | --- |
| SFA | −0.038[−0.088; 0.017] |
| MUFA | 0.024[−0.101; 0.147] |
| PUFA | 0.015[−0.099; 0.132] |
| PUFAn3 | 0.063[−0.077; 0.204] |
| PUFAn6 | −0.062[−0.204; 0.081] |
| ACL | 0.307[0.049; 0.546] |
| DBI | 0.260[0.004; 0.493] |
| PI | 0.236[−0.023; 0.476] |
| AI | −0.228[−0.531; 0.078] |
| C14.0 | −0.209[−0.349; −0.079] |
| C14.1 | −0.002[−0.123; 0.123] |
| C16.0 | −0.052[−0.140; 0.039] |
| C16.1.n7 | −0.064[−0.264; 0.117] |
| C18.0 | 0.029[−0.032; 0.092] |
| C18.1.n9 | 0.016[−0.122; 0.146] |
| C18.2.n6 | −0.151[−0.343; 0.045] |
| C18.3.n3 | 0.094[−0.244; 0.447] |
| C18.4.n3 | 0.052[−0.052; 0.156] |
| C20.0 | 0.056[−0.038; 0.149] |
| C20.1.n9 | 0.116[0.005; 0.232] |
| C20.2.n6 | 0.076[−0.039; 0.202] |
| C20.3.n6 | −0.013[−0.114; 0.095] |
| C20.4.n6 | 0.169[0.040; 0.289] |
| C20.5.n3 | 0.058[−0.088; 0.209] |
| C22.0 | −0.023[−0.144; 0.094] |
| C22.1.n9 | 0.022[−0.139; 0.194] |
| C22.4.n6 | 0.294[0.159; 0.419] |
| C22.5.n6 | 0.123[−0.054; 0.313] |
| C22.5.n3 | 0.072[−0.126; 0.261] |
| C22.6.n3 | 0.022[−0.146; 0.189] |
| C24.0 | −0.012[−0.122; 0.103] |
| C24.1.n9 | 0.042[−0.062; 0.142] |
| C24.5.n3 | −0.047[−0.164; 0.066] |
| C24.6.n3 | 0.053[−0.042; 0.149] |

**Table S2**. Average marginal effects from lifespan models. Values are effect estimates from Bayesian phylogenetic models with 95% credible intervals.

| **Response** | **Body mass** | **Lifespan** | **Migration distance** |
| --- | --- | --- | --- |
| SFA | −0.011[−0.089; 0.071] | −0.015[−0.079; 0.049] | 0.043[0.003; 0.083] |
| MUFA | 0.018[−0.151; 0.186] | −0.023[−0.156; 0.112] | −0.034[−0.119; 0.051] |
| PUFA | 0.000[−0.159; 0.159] | 0.011[−0.117; 0.136] | −0.018[−0.096; 0.062] |
| PUFAn3 | 0.111[−0.075; 0.298] | −0.032[−0.182; 0.122] | 0.059[−0.039; 0.154] |
| PUFAn6 | −0.113[−0.302; 0.076] | 0.032[−0.116; 0.184] | −0.059[−0.156; 0.039] |
| ACL | 0.025[−0.354; 0.383] | 0.349[0.045; 0.658] | −0.076[−0.269; 0.119] |
| DBI | 0.061[−0.324; 0.419] | 0.224[−0.099; 0.555] | −0.071[−0.268; 0.132] |
| PI | 0.005[−0.371; 0.373] | 0.283[−0.038; 0.607] | −0.018[−0.224; 0.183] |
| AI | −0.165[−0.565; 0.239] | −0.071[−0.384; 0.243] | 0.018[−0.179; 0.220] |
| C14.0 | −0.173[−0.365; 0.016] | −0.013[−0.182; 0.161] | 0.101[−0.002; 0.201] |
| C14.1 | 0.055[−0.142; 0.252] | −0.069[−0.246; 0.116] | −0.022[−0.141; 0.093] |
| C16.0 | 0.017[−0.107; 0.146] | −0.064[−0.164; 0.036] | 0.046[−0.015; 0.107] |
| C16.1.n7 | −0.007[−0.256; 0.233] | −0.024[−0.232; 0.202] | 0.118[−0.027; 0.249] |
| C18.0 | 0.003[−0.09; 0.092] | 0.036[−0.037; 0.110] | 0.001[−0.044; 0.047] |
| C18.1.n9 | 0.034[−0.146; 0.210] | −0.064[−0.204; 0.072] | −0.047[−0.139; 0.042] |
| C18.2.n6 | −0.106[−0.362; 0.150] | −0.063[−0.252; 0.133] | −0.005[−0.129; 0.119] |
| C18.3.n3 | 0.452[0.076; 0.842] | −0.401[−0.688; −0.109] | 0.218[0.031; 0.405] |
| C18.4.n3 | 0.062[−0.111; 0.232] | −0.009[−0.168; 0.151] | 0.006[−0.095; 0.103] |
| C20.0 | −0.027[−0.177; 0.118] | 0.116[−0.027; 0.258] | −0.015[−0.105; 0.071] |
| C20.1.n9 | −0.009[−0.182; 0.155] | 0.176[0.011; 0.338] | −0.067[−0.175; 0.035] |
| C20.2.n6 | 0.013[−0.151; 0.179] | 0.082[−0.065; 0.227] | −0.025[−0.120; 0.069] |
| C20.3.n6 | −0.026[−0.19; 0.136] | 0.021[−0.118; 0.164] | 0.015[−0.074; 0.103] |
| C20.4.n6 | 0.066[−0.105; 0.229] | 0.132[−0.006; 0.267] | −0.017[−0.107; 0.070] |
| C20.5.n3 | 0.161[−0.102; 0.434] | −0.084[−0.307; 0.146] | 0.008[−0.135; 0.143] |
| C22.0 | −0.175[−0.366; 0.006] | 0.175[0.004; 0.348] | −0.090[−0.198; 0.014] |
| C22.1.n9 | 0.015[−0.254; 0.328] | 0.141[−0.102; 0.384] | 0.057[−0.103; 0.231] |
| C22.4.n6 | 0.203[0.036; 0.365] | 0.175[0.021; 0.326] | 0.069[−0.036; 0.167] |
| C22.5.n6 | −0.001[−0.246; 0.255] | 0.126[−0.083; 0.331] | −0.072[−0.218; 0.073] |
| C22.5.n3 | 0.040[−0.211; 0.282] | 0.107[−0.094; 0.309] | 0.119[−0.017; 0.243] |
| C22.6.n3 | −0.147[−0.379; 0.089] | 0.189[0.005; 0.377] | −0.050[−0.172; 0.069] |
| C24.0 | −0.074[−0.261; 0.114] | 0.073[−0.097; 0.243] | −0.040[−0.151; 0.067] |
| C24.1.n9 | −0.060[−0.236; 0.108] | 0.123[−0.035; 0.285] | −0.032[−0.136; 0.069] |
| C24.5.n3 | −0.136[−0.332; 0.056] | 0.094[−0.089; 0.278] | −0.065[−0.179; 0.045] |
| C24.6.n3 | 0.049[−0.101; 0.195] | 0.012[−0.120; 0.148] | −0.013[−0.106; 0.075] |

**Table S3**. Average marginal effects from annual fecundity models. Values are effect estimates from Bayesian phylogenetic models with 95% credible intervals.

| **Response** | **Body mass** | **Annual fecundity** | **Migration distance** |
| --- | --- | --- | --- |
| SFA | −0.041[−0.099; 0.025] | −0.028[−0.077; 0.019] | 0.038[−0.001; 0.078] |
| MUFA | 0.034[−0.119; 0.178] | 0.055[−0.048; 0.163] | −0.034[−0.121; 0.050] |
| PUFA | −0.003[−0.137; 0.133] | −0.019[−0.116; 0.079] | −0.021[−0.101; 0.059] |
| PUFAn3 | 0.081[−0.078; 0.239] | −0.011[−0.131; 0.109] | 0.059[−0.038; 0.155] |
| PUFAn6 | −0.080[−0.242; 0.084] | 0.011[−0.111; 0.131] | −0.059[−0.156; 0.039] |
| ACL | 0.261[−0.042; 0.546] | −0.052[−0.292; 0.197] | −0.071[−0.275; 0.131] |
| DBI | 0.254[−0.044; 0.542] | 0.012[−0.226; 0.256] | −0.045[−0.252; 0.159] |
| PI | 0.227[−0.079; 0.525] | −0.027[−0.272; 0.226] | 0.009[−0.195; 0.214] |
| AI | −0.282[−0.636; 0.082] | −0.101[−0.347; 0.145] | −0.001[−0.198; 0.196] |
| C14.0 | −0.196[−0.347; −0.048] | −0.009[−0.140; 0.119] | 0.088[−0.015; 0.185] |
| C14.1 | −0.012[−0.159; 0.136] | −0.010[−0.147; 0.133] | −0.037[−0.154; 0.079] |
| C16.0 | −0.038[−0.141; 0.067] | −0.007[−0.083; 0.068] | 0.045[−0.017; 0.104] |
| C16.1.n7 | 0.083[−0.109; 0.258] | 0.165[0.006; 0.324] | 0.158[0.021; 0.281] |
| C18.0 | 0.011[−0.059; 0.080] | −0.035[−0.088; 0.019] | −0.004[−0.048; 0.039] |
| C18.1.n9 | 0.023[−0.137; 0.179] | 0.062[−0.049; 0.173] | −0.046[−0.139; 0.043] |
| C18.2.n6 | −0.196[−0.423; 0.033] | −0.062[−0.209; 0.083] | −0.024[−0.149; 0.102] |
| C18.3.n3 | 0.230[−0.153; 0.625] | 0.039[−0.230; 0.306] | 0.219[0.021; 0.427] |
| C18.4.n3 | 0.079[−0.046; 0.204] | 0.045[−0.069; 0.163] | 0.009[−0.090; 0.106] |
| C20.0 | 0.037[−0.079; 0.146] | −0.026[−0.132; 0.079] | −0.019[−0.109; 0.069] |
| C20.1.n9 | 0.034[−0.099; 0.167] | −0.113[−0.235; 0.015] | −0.093[−0.197; 0.011] |
| C20.2.n6 | 0.075[−0.068; 0.236] | 0.012[−0.106; 0.139] | −0.032[−0.131; 0.064] |
| C20.3.n6 | 0.056[−0.064; 0.192] | 0.112[0.007; 0.222] | 0.042[−0.048; 0.127] |
| C20.4.n6 | 0.177[0.026; 0.327] | 0.023[−0.093; 0.141] | −0.007[−0.099; 0.081] |
| C20.5.n3 | 0.044[−0.137; 0.227] | −0.022[−0.176; 0.133] | −0.020[−0.159; 0.114] |
| C22.0 | −0.065[−0.205; 0.072] | −0.027[−0.151; 0.102] | −0.095[−0.204; 0.009] |
| C22.1.n9 | −0.012[−0.215; 0.216] | −0.063[−0.232; 0.111] | −0.020[−0.173; 0.133] |
| C22.4.n6 | 0.361[0.203; 0.528] | 0.069[−0.069; 0.219] | 0.076[−0.034; 0.184] |
| C22.5.n6 | 0.134[−0.087; 0.376] | 0.061[−0.111; 0.245] | −0.062[−0.209; 0.087] |
| C22.5.n3 | 0.165[−0.041; 0.374] | 0.058[−0.095; 0.216] | 0.143[0.012; 0.267] |
| C22.6.n3 | −0.024[−0.218; 0.179] | −0.056[−0.204; 0.097] | −0.042[−0.159; 0.075] |
| C24.0 | −0.029[−0.166; 0.109] | −0.018[−0.139; 0.104] | −0.039[−0.147; 0.068] |
| C24.1.n9 | 0.020[−0.105; 0.146] | −0.026[−0.142; 0.089] | −0.029[−0.134; 0.069] |
| C24.5.n3 | −0.078[−0.223; 0.061] | −0.029[−0.162; 0.104] | −0.063[−0.178; 0.047] |
| C24.6.n3 | 0.057[−0.059; 0.174] | 0.017[−0.090; 0.123] | −0.017[−0.112; 0.073] |

**Table S4**. Average marginal effects from basal metabolic rate models. Values are effect estimates from Bayesian phylogenetic models with 95% credible intervals.

| **Response** | **Body mass** | **BMR** |
| --- | --- | --- |
| SFA | −0.098[−0.316; 0.119] | 0.052[−0.153; 0.257] |
| MUFA | 0.525[0.038; 1.029] | −0.432[−0.879; 0.006] |
| PUFA | −0.441[−0.951; 0.048] | 0.409[−0.019; 0.842] |
| PUFAn3 | −0.084[−0.639; 0.472] | 0.150[−0.359; 0.652] |
| PUFAn6 | 0.085[−0.465; 0.666] | −0.149[−0.660; 0.345] |
| ACL | −0.643[−1.965; 0.552] | 0.925[−0.173; 2.084] |
| DBI | −0.737[−1.998; 0.401] | 0.924[−0.127; 2.043] |
| PI | −0.925[−2.110; 0.231] | 1.115[−0.004; 2.252] |
| AI | −0.611[−1.677; 0.427] | 0.154[−0.826; 1.144] |
| C14.0 | 0.024[−0.559; 0.593] | −0.184[−0.721; 0.364] |
| C14.1 | −0.149[−0.805; 0.499] | 0.196[−0.423; 0.830] |
| C16.0 | 0.027[−0.322; 0.392] | −0.069[−0.381; 0.235] |
| C16.1.n7 | 0.404[−0.461; 1.255] | −0.447[−1.228; 0.316] |
| C18.0 | −0.109[−0.368; 0.139] | 0.124[−0.099; 0.353] |
| C18.1.n9 | 0.575[0.068; 1.075] | −0.486[−0.946; −0.029] |
| C18.2.n6 | −0.381[−1.064; 0.303] | 0.066[−0.528; 0.654] |
| C18.3.n3 | 0.017[−1.155; 1.156] | −0.021[−1.012; 0.983] |
| C18.4.n3 | −0.022[−0.632; 0.575] | 0.059[−0.503; 0.632] |
| C20.0 | −0.126[−0.632; 0.369] | 0.160[−0.314; 0.642] |
| C20.1.n9 | −0.043[−0.624; 0.522] | 0.151[−0.389; 0.703] |
| C20.2.n6 | −0.264[−0.819; 0.284] | 0.294[−0.228; 0.836] |
| C20.3.n6 | −0.032[−0.504; 0.442] | 0.025[−0.423; 0.472] |
| C20.4.n6 | 0.124[−0.599; 0.351] | 0.341[−0.075; 0.759] |
| C20.5.n3 | −0.122[−0.989; 0.805] | 0.278[−0.527; 1.106] |
| C22.0 | −0.260[−0.871; 0.338] | 0.206[−0.360; 0.788] |
| C22.1.n9 | 0.053[−1.039; 1.096] | 0.166[−0.732; 1.149] |
| C22.4.n6 | 0.129[−0.422; 0.651] | 0.155[−0.336; 0.669] |
| C22.5.n6 | −0.346[−1.114; 0.422] | 0.429[−0.299; 1.146] |
| C22.5.n3 | −0.155[−0.944; 0.643] | 0.288[−0.411; 0.983] |
| C22.6.n3 | −0.437[−1.121; 0.208] | 0.380[−0.241; 1.032] |
| C24.0 | −0.196[−0.819; 0.417] | 0.176[−0.423; 0.777] |
| C24.1.n9 | 0.058[−0.562; 0.644] | −0.052[−0.618; 0.549] |
| C24.5.n3 | −0.352[−1.026; 0.267] | 0.277[−0.318; 0.906] |
| C24.6.n3 | −0.158[−0.656; 0.317] | 0.229[−0.234; 0.707] |
